# SIRT1 and AROS suppress doxorubicin-induced apoptosis via inhibition of GSK3β activity in neuroblastoma cells

**DOI:** 10.1080/19768354.2020.1726461

**Published:** 2020-02-12

**Authors:** Jeong Woo Kim, Ji Hye Yang, Eun-Joo Kim

**Affiliations:** Department of Molecular Biology, Dankook University, Cheonan-si, Korea

**Keywords:** SIRT1, GSK3β, apoptosis, neuroblastoma

## Abstract

SIRT1, the best-characterized member of the sirtuin family of deacetylases, is involved in cancer, apoptosis, inflammation, and metabolism. Active regulator of SIRT1 (AROS) was the first identified direct regulator of SIRT1. An increasing number of reports have indicated that SIRT1 plays an important role in controlling brain tumors. Here, we demonstrated that depletion of SIRT1 and AROS increases doxorubicin-mediated apoptosis in human neuroblastoma SH-SY5Y cells. Glycogen synthase kinase 3β (GSK3β) promoted doxorubicin-mediated apoptosis, but this effect was abolished by overexpression of SIRT1 and AROS. Interestingly, SIRT1 and AROS interacted with GSK3β and increased inhibitory phosphorylation of GSK3β on Ser9. Finally, we determined that AROS cooperates with SIRT1 to suppress GSK3β acetylation. Taken together, our results suggest that SIRT1 and AROS inhibit GSK3β activity and provide additional insight into drug resistance in the treatment of neuroblastoma.

## Introduction

Neuroblastoma is one of the most lethal brain tumor in children, which is largely due to a lack of response to current therapies (Mischel et al. 2004; Löscher and Potschka 2005). Doxorubicin (DOX), the first anthracycline isolated, has been widely used as an effective anticancer drug for many types of cancers including breast cancer, soft tissue sarcomas, and hematological malignancies (Minotti et al. 2004; Lopes et al. 2008). Although DOX has been used as a routine chemotherapeutic agent, it induces drug resistance and exhibits poor penetration across the blood–brain barrier when used in the treatment of brain tumors (Zhang et al. 2013). However, DOX resistance in neuroblastoma has been poorly studied.

SIRT1, the well-characterized mammalian homolog of yeast Sir2, is an NAD^+^-dependent deacetylase involved in lifespan extension, cancer, inflammation, and metabolism (Herskovits and Guarente 2014; Yang et al. 2015). Active regulator of SIRT1 (AROS) was the first identified direct regulator of SIRT1 (Kim et al. 2007). Recently, an increasing number of reports have shown that SIRT1 plays an important role in brain tumors. SIRT1 is overexpressed in brain metastases of non-small cell lung cancer (NSCLC) and positively regulates the migration of NSCLC cells (Han et al. 2013). In human medulloblastoma cells, SIRT1 expression patterns have also been correlated with their formation and prognosis (Ma et al. 2013). Furthermore, deficiency of miR-34a targeting SIRT1 accelerates medulloblastoma formation (Thor et al. 2015), and its mimics induced cell death in p53-mutated medulloblastoma and glioblastoma cells (Fan et al. 2014).

Glycogen synthase kinase 3β (GSK3β) is a serine/threonine kinase that was originally identified as a key enzyme in glycogen synthesis (Embi et al. 1980; Kaidanovich-Beilin and Woodgett 2011). GSK3β is also a downstream protein of various signaling pathways, including the Wnt/β-catenin and phosphoinositide 3 kinase (PI3K) pathways, which are involved in cell proliferation and differentiation in many tissues (Tejeda-Muñoz and Robles-Flores 2015; Hermida et al. 2017). Recent studies have shown that GSK3β is overexpressed in several cancer cell types, and inhibition of GSK3β decreased cancer cell proliferation and survival (Luo 2009; Zeng et al. 2014; Domoto et al. 2016). Whereas, several studies have reported that GSK3β promotes apoptosis in various stress conditions (Bijur et al. 2000; Song et al. 2002; Watcharasit et al. 2002). Therefore, further study on the role of GSK3β in cancer is still needed. Furthermore, the functional correlation between SIRT1 and GSK3β in brain tumorigenesis is unknown.

Here, we investigated the role of SIRT1/AROS in DOX-mediated cytotoxicity and identified GSK3β as a novel SIRT1/AROS interacting protein. Our results suggest that SIRT1 and AROS suppress GSK3β activity and acetylation, regulating the chemoresistance of doxorubicin in neuroblastoma.

## Materials and methods

### Plasmids and cloning

Full-length GSK3β was PCR amplified, subcloned into Flag-tagged pcDNA3 and pEGFP-C3 vectors (BD Biosciences), and verified by sequencing. Other constructs were generated as previously described (Kim et al. 2007).

### Cell culture and transfection

Human neuroblastoma SH-SY5Y cells were maintained in a minimal essential medium. HEK293 cells were maintained in Dulbecco’s modified Eagle’s medium containing 10% fetal bovine serum and an antibiotic–antimycotic mix (all from Invitrogen). Cells were grown in a humidified incubator at 37°C with 5% CO_2_. Under experimental conditions, plasmids were transfected using Lipofectamine with Plus Reagent (Invitrogen) or Polyethylenimine (PEI, Polysciences Inc.) according to the manufacturer’s instructions. To assess the effects of GSK3β, cells were treated with 10 μM SB216763 (Sigma-Aldrich) for 2 h prior to exposure to 0.1 μM DOX for 12 h.

### Immunoprecipitation and western blotting

Immunoprecipitation (IP) and western blotting (WB) were conducted as previously described (Cho et al. 2006). Briefly, transfected cells were lysed in cold lysis buffer and incubated with 1:200 dilutions of appropriate antibodies overnight at 4°C. The lysates were further incubated with 25 µL of protein A/G Plus agarose beads for 4 h (Santa Cruz Biotechnology). After incubation, IP complexes were washed and resuspended in loading buffer. Boiled samples were analyzed using 8–12% sodium dodecyl sulfate-polyacrylamide gel electrophoresis and transferred to nitrocellulose membranes. The membranes were incubated with the following antibodies: GSK3α/β (sc-7291), poly (ADP-ribose) polymerase-1 (PARP-1; sc-8007), green fluorescent protein (GFP; sc-8334), and β-actin (sc-4778) (all from Santa Cruz Biotechnology); pS9-GSK3β (#9336) and Ac-K2-100 (#9814) (Cell Signaling Technology); Flag M2 (F3165; Sigma-Aldrich); and Myc (05-724; Millipore). The protein bands were developed using an enhanced chemiluminescence system (iNtRON Biotechnology).

### Flow cytometry

Flow cytometry was performed as previously described (Kim et al. 2002). At 12 h after exposure to DOX, adherent and detached cells were collected and fixed in 70% ethanol. Cells were stained with 5 μg/mL propidium iodine-containing 100 μg/mL RNase A for 30 min. A total of 1 × 10^6^ cells were analyzed with a flow cytometer (Guava EasyCyte, Cell Signaling Technology).

### Hoechst staining

DOX-treated cells were fixed in 70% ethanol for 10 min at room temperature and stained with 1 mg/mL Hoechst (Sigma-Aldrich) at a dilution of 1:1000 for 10 min. Apoptotic cells were observed under an immunofluorescence microscope (Carl Zeiss).

### Confocal microscopy

Experiments were performed as previously described (Kim et al. 2002). SH-SY5Y cells were seeded in a culture chamber and transfected with GFP-GSK3β, Flag-SIRT1, and Myc-AROS. Cells were washed with phosphate-buffered saline (PBS) and fixed in 4% paraformaldehyde for 10 min at room temperature. Following permeabilization with 0.3% Triton X-100 for 15 min, cells were blocked in PBS containing 3% bovine serum albumin for 1 h. Cells were then incubated with rabbit polyclonal anti-SIRT1 antibody (sc-15404; Santa Cruz Biotechnology) and mouse monoclonal anti-Myc antibody. Cells were followed by incubation with rhodamine-conjugated anti-rabbit IgG and DyLight 405-conjugated anti-mouse IgG (Jackson ImmunoResearch) for 1 h. Mounted cells were visualized with a confocal microscope (Leica).

### RNA interference

The sequences of the shRNA duplexes targeting SIRT1 or AROS were as follows: SIRT1, ACTTTGCTGTAACCCTGTA and AROS, GTTTCTGACCAGGACGAGA. shRNA transfection was performed using Lipofectamine with Plus Reagent (Invitrogen) according to the manufacturer’s instructions. Knockdown was verified by WB with the appropriate antibodies (Santa Cruz Biotechnology), or was analyzed by PCR using pair of primers: for the *SIRT1* coding sequence; forward, 5′-TCGCAACTATACCCAGAACATAGACA-3′ and reverse, 5′-CTGTTGCAAAGGAACCATGACA-3′: for the *AROS* coding sequence; forward, 5′-GGAAGACGAAGGCAATTCAGGC-3′ and reverse, 5′-TCGGTGAACACGGTGCC-3′: for control *GAPDH* coding sequence; forward, 5′-CTGCACCACCAACTGCTTAGC-3′ and reverse, 5′-GGGCCATCCACAGTCTTCTGG-3′.

## Results

### Depletion of SIRT1 and AROS increase DOX-induced apoptosis

**Figure 1.**
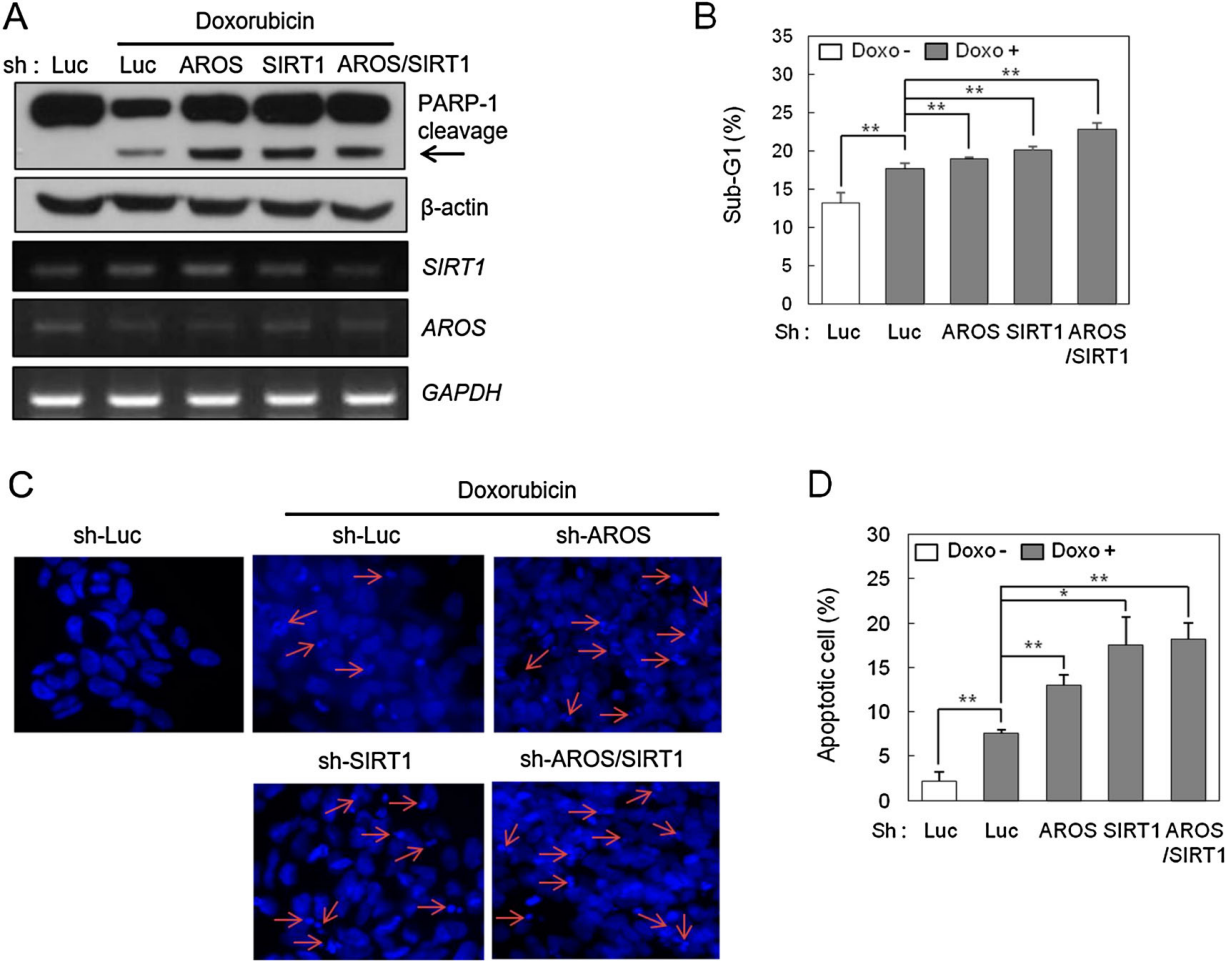
SIRT1 and AROS deficiency increase doxorubicin (DOX)-induced cytotoxicity. SH-SY5Y cells were transfected with sh-Luciferase (sh-Luc), sh-SIRT1, and sh-AROS. At 48 h after transfection, cells were treated with 0.1 μM DOX for 12 h. (A) Cleaved PARP-1 was detected by western blotting (WB; arrow). Knockdown efficiency was measured using RT-PCR with SIRT1 or AROS primers. *GAPDH* was used as an internal control. (B) SH-SY5Y Cells were fixed in 70% ethanol and stained with propidium iodide, and the Sub-G1 fraction was analyzed by flow cytometry. Quantification of the Sub-G1 population in response to DOX. Each value represents the mean ± standard deviation (SD) of three independent experiments (**P < 0.01). (C) Chromatin condensation and nuclear fragmentation as detected by Hoechst staining. (D) Quantification of apoptotic cell death in response to DOX. The percentage of apoptotic cells was calculated. All data are expressed as the mean ± SD of three independent experiments (*P < 0.05, **P < 0.01).

Based on recent reports on the role of SIRT1 in brain tumors, we first explored the effects of SIRT1 on apoptosis induced by DOX in human neuroblastoma SH-SY5Y cells. As shown in Figure 1(A), the shRNA-mediated knockdown of SIRT1 significantly increased apoptosis compared to sh-Luciferase-transfected cells as indicated by PARP-1 cleavage, a marker of apoptosis. AROS deficiency or depletion of both SIRT1 and AROS also effectively enhanced PARP-1 cleavage (Figure 1(A)). Flow cytometry revealed that the sub-G1 phase population, an indicator of apoptotic cell death, was increased in SIRT1-deficient cells. Similarly, knockdown of AROS or both SIRT1 and AROS also enhanced the percentage of cells in sub-G1 (Figure 1(B)). Furthermore, Hoechst staining revealed that chromatin condensation and nuclear fragmentation were markedly increased following depletion of SIRT1 and/or AROS (Figure 1(C and D)). Overall, our data suggest that deficiency of SIRT1 and/or AROS increased cellular sensitivity to DOX-induced apoptosis and the effects of AROS may be achieved through activation of SIRT1.

### SIRT1 and AROS decrease GSK3β-mediated sensitivity to DOX

**Figure 2.**
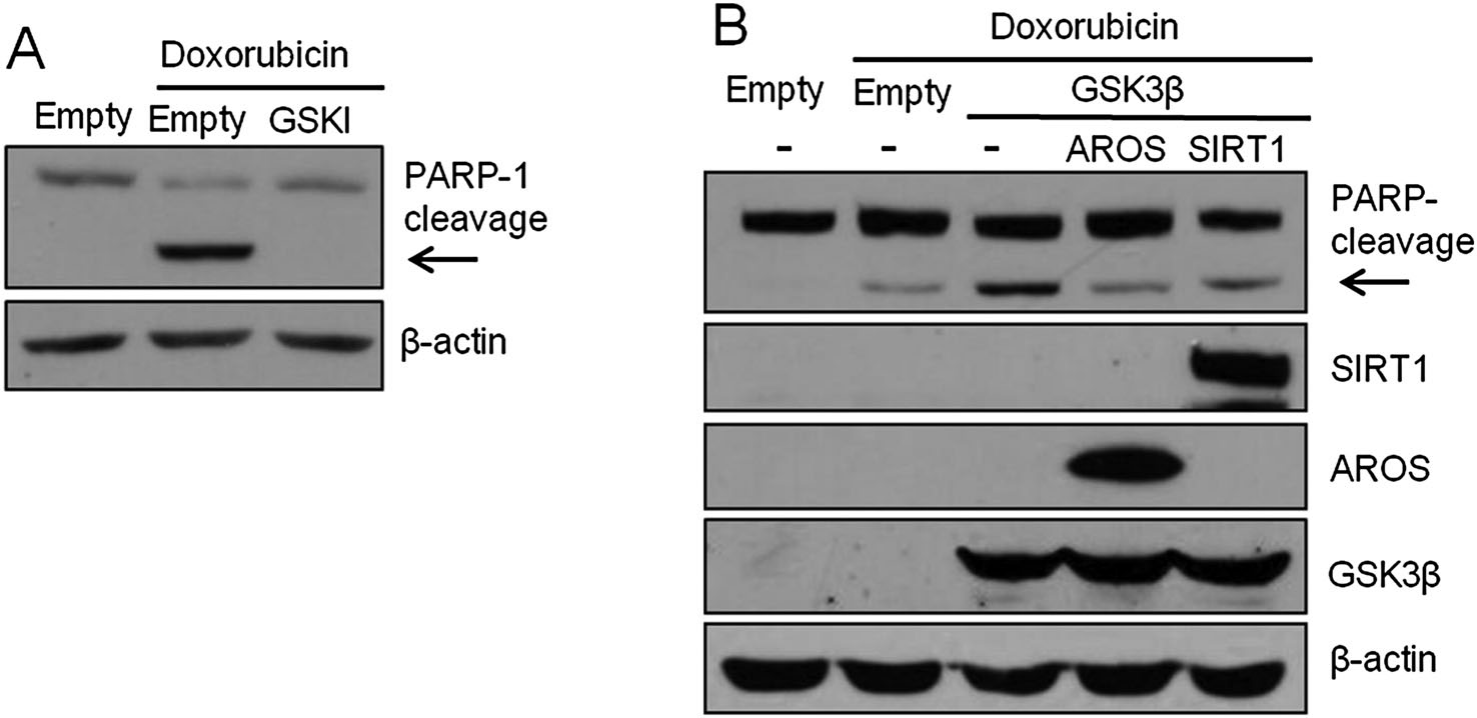
SIRT1 and AROS abolish the effects of GSK3β on DOX-induced apoptosis. (A) SH-SY5Y cells were exposed to 0.1 μM DOX for 12 h. Cells were treated with 10 μM SB216763 2 h before DOX treatment. PARP-1 cleavage was detected by WB (arrow). (B) SH-SY5Y cells were co-transfected with GFP-GSK3β and Flag-AROS or Flag-SIRT1. At 24 h after transfection, cells were treated with 0.1 μM DOX for 12 h. Cleaved PARP-1 was observed by WB (arrow).

A recent study reported that SIRT1 promotes axonogenesis by deacetylating Akt and inactivating GSK3β (Li et al. 2013). Inhibition of GSK3β attenuates stress-induced apoptosis in SH-SY5Y cells (Bijur et al. 2000; Song et al. 2002). To investigate the functional relevance between GSK3β and SIRT1 in response to DNA damage, we first determined the effects of GSK3β inhibition on apoptosis followed by DOX treatment in SH-SY5Y cells. As expected, treatment with SB216763, a pharmacological inhibitor of GSK3β, greatly attenuated PARP-1 cleavage induced by DOX (Figure 2(A)). Next, we addressed the effects of GSK3β following DOX treatment. As shown in Figure 2(B), overexpression of GSK3β markedly enhanced DOX-induced PARP-1 cleavage. By contrast, SIRT1 significantly impaired GSK3β-mediated sensitivity to DOX (Figure 2(B)). Furthermore, PARP-1 cleavage demonstrated a similar resistance effect of AROS expression on GSK3β-induced apoptosis (Figure 2(B)). These results suggest the roles of SIRT1 and AROS in DOX resistance and further support the role of GSK3β function in DOX sensitivity.

### SIRT1 and AROS interact with GSK3β in the nucleus

**Figure 3.**
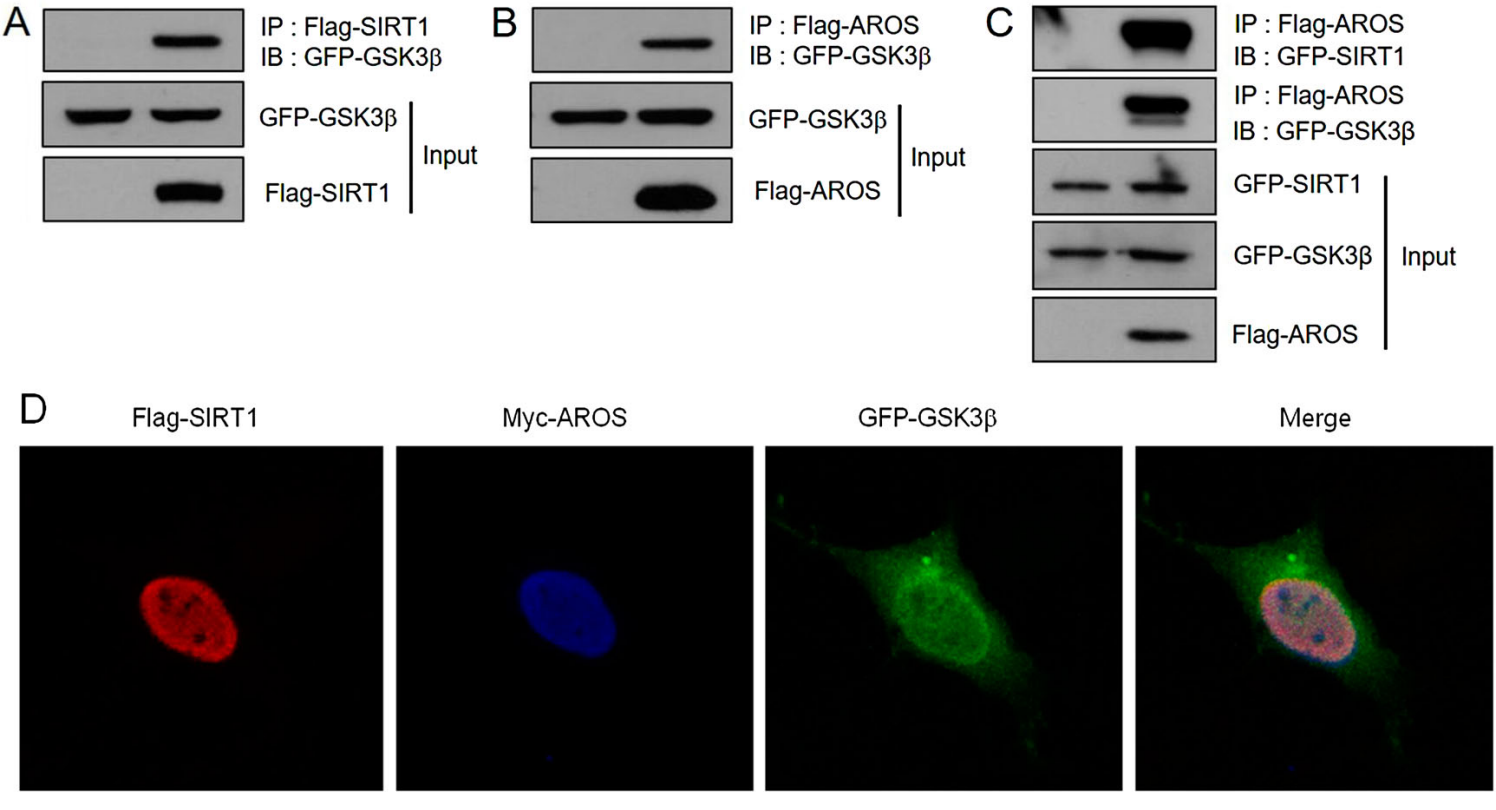
SIRT1 and AROS associate with GSK3β. For immunoprecipitation (IP) assay, HEK293 cells were co-transfected with GFP-GSK3β and empty vector or Flag-SIRT1. Interaction was determined by IP with anti-Flag antibody and subsequent WB with anti-GFP antibody. (B) HEK293 cells were transfected with GFP-GSK3β and empty vector or Flag-AROS. IP was performed with anti-Flag antibody and bound protein was examined with anti-GFP antibody. (C) HEK293 cells were transfected with GFP-GSK3β, GFP-SIRT1, and empty vector or Flag-AROS. Cell lysates were immunoprecipitated with anti-Flag antibody and bound protein was examined with anti-GFP antibody. (D) SH-SY5Y cells were transfected with GFP-GSK3β, Flag-SIRT1, and Myc-AROS. Cells were then incubated with rabbit polyclonal anti-SIRT1 antibody and mouse monoclonal anti-Myc antibody. Cellular location was observed by rhodamine-conjugated anti-rabbit IgG and DyLight 405-conjugated anti-mouse IgG using confocal microscopy

To examine whether SIRT1 physically interacts with GSK3β, we co-transfected HEK293 cells with empty vector or Flag-tagged SIRT1 and GFP-tagged GSK3β. IP using an anti-Flag antibody and subsequent WB with anti-GFP antibody demonstrated that SIRT1 interacts with GSK3β (Figure 3(A)). Co-transfection followed by IP indicated that AROS also associates with GSK3β (Figure 3(B)). Next, we confirmed the complex formation of these proteins. GFP-SIRT1 and GFP-GSK3β were detected by IP with anti-Flag antibody for AROS, supporting GSK3β forms the complex with SIRT1 and AROS (Figure 3(C)). We previously reported that SIRT1 and AROS primarily colocalize in the nucleus (Kim et al. 2007), whereas GSK3β is predominantly found in the cytoplasm. To further substantiate the interaction, we observed the subcellular distribution of SIRT1, AROS, and GSK3β in SH-SY5Y cells. As shown in Figure 3(D), confocal microscopy revealed that Flag-SIRT1, Myc-AROS, and GFP-GSK3β colocalized in the nucleus. Overall, our data suggest that SIRT1 and AROS physically interact with GSK3β in the nucleus, and may perturb its activity and cytoplasmic function.

### SIRT1 and AROS increase inhibitory phosphorylation of GSK3β

**Figure 4.**
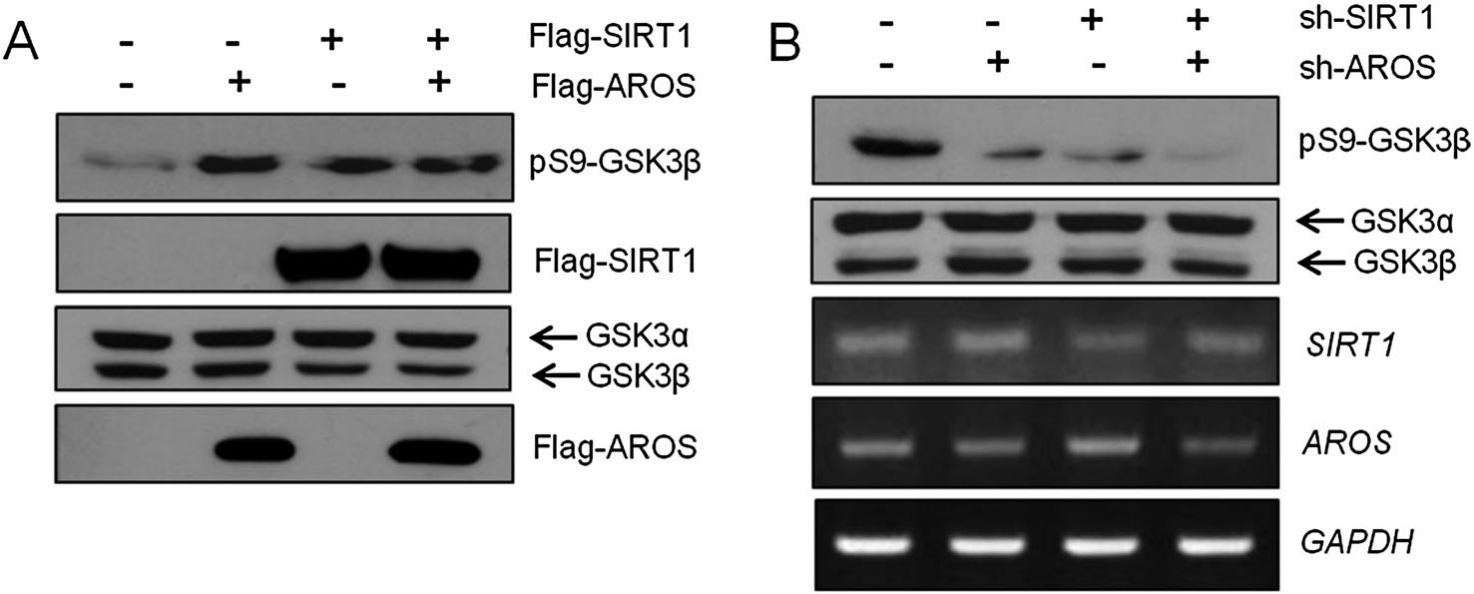
Effects of SIRT1 and AROS on the phosphorylation of GSK3β. (A) HEK293 cells were transiently transfected with Flag-SIRT1 and/or Flag-AROS. Phosphorylation of GSK3β at Ser9 was monitored by anti-pS9-GSK3β antibody. (B) SH-SY5Y cells were transfected with sh-Luc, sh-SIRT1, or sh-AROS. Lysates were subjected to WB with anti-pS9-GSK3β antibody. Knockdown efficiency was measured using RT-PCR with SIRT1 or AROS primers. *GAPDH* was used as an internal control.

To explore the molecular mechanism underlying the regulation of GSK3β activity by SIRT1 and AROS, we analyzed the effects of both SIRT1 and AROS on the inhibitory phosphorylation of GSK3β. GSK3β activity is inhibited by phosphorylation of Ser9 via p70 S6 kinase, p90Rsk, and Akt (Ali et al. 2001; Mulholland et al. 2006). As shown in Figure 4(A), overexpression of SIRT1 or AROS strongly increased phosphorylation of GSK3β Ser9 in HEK293 cells. By contrast, SIRT1 or AROS depletion resulted in markedly decreased phosphorylation of GSK3β Ser9 in SH-SY5Y cells (Figure 4(B)). These results demonstrate that SIRT1 and AROS inhibit DOX-induced apoptosis by suppressing the activity of GSK3β.

### SIRT1 deacetylates GSK3β by cooperating with AROS

**Figure 5.**
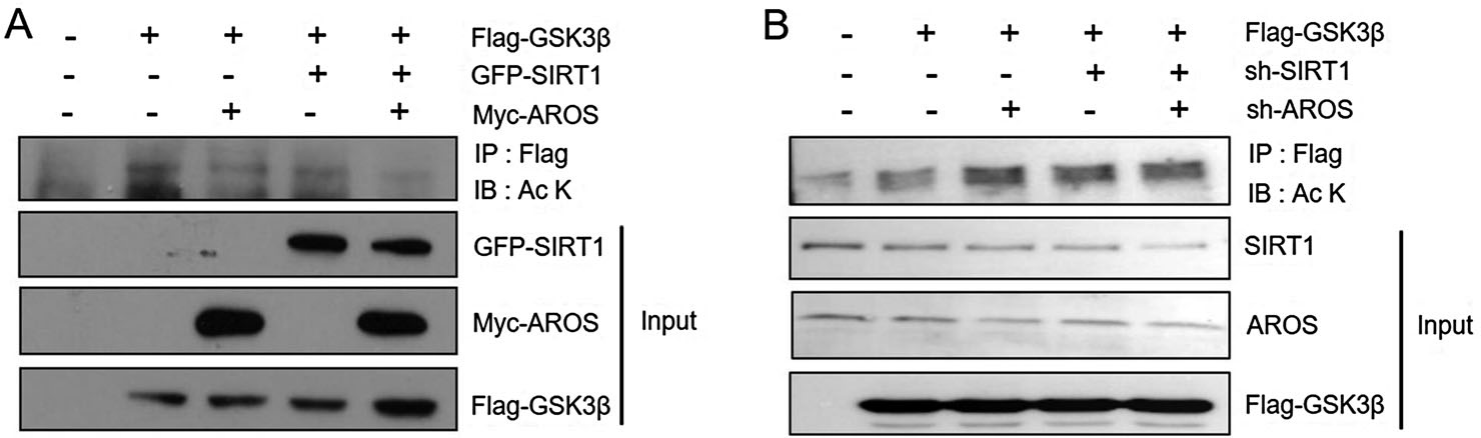
Effects of SIRT1 and AROS on deacetylation of GSK3β. (A) HEK293 cells were co-transfected with Flag-GSK3β and GFP-SIRT1 or Myc-AROS. Cell lysates were subjected to IP with anti-Flag antibody and analyzed by WB with anti-acetyl lysine antibody. (B) HEK293 cells were transfected with sh-Luc, sh-SIRT1, or sh-AROS. Cell lysates were subjected to IP with anti-Flag antibody and analyzed by WB with anti-acetyl lysine antibody.

To investigate the possibility of GSK3β as a SIRT1 substrate, we assessed whether SIRT1 deacetylates GSK3β. HEK293 cells were co-transfected with Flag-tagged GSK3β and GFP-tagged SIRT1 and/or Myc-tagged AROS. Anti-Flag immunoprecipitates probed with anti-acetyl lysine antibody revealed that the level of GSK3β acetylation was substantially reduced by SIRT1 and/or AROS expression (Figure 5(A)). By contrast, SIRT1 or AROS depletion resulted in significantly increased the level of Flag-GSK3β acetylation in HEK293 cells (Figure 5(B)). These results suggest that SIRT1 promotes deacetylation of GSK3β, likely through cooperation with AROS, and may inhibit its role in DOX-induced apoptosis in neuroblastoma cells.

## Discussion

Neuroblastoma is the most common type of extracranial solid tumor in children and is responsible for up to 15% of all pediatric oncologic deaths (Whittle et al. 2017; Swift et al. 2018). Neuroblastoma is a heterogeneous tumor with a wide range of clinical behaviors; some tumors exhibit aggressive growth and are more likely to metastasize (Maris 2010). However, the development of therapeutic approaches has been limited despite decades of intensive research and clinical trials. Understanding the molecular mechanisms of chemotherapy resistance in neuroblastoma is critical for improving the poor prognosis.

SIRT1 is an NAD^+^-dependent deacetylase that plays a crucial role in tumorigenesis through the deacetylation of tumor regulatory proteins (Cheng et al. 2013). Recent studies have shown that suppression of deacetylase activity or expression of SIRT1 induces apoptosis in human neuroblastoma cells (Tu et al. 2018; Fu et al. 2019). These reports suggest that inhibition of SIRT1 might be a promising target in the treatment of neuroblastoma. GSK3β is also an interesting potential target for neuroblastoma therapy, as it has been implicated in cell growth inhibition and apoptosis in various cancers. GSK3β promotes apoptosis in various damage conditions (Bijur et al. 2000; Song et al. 2002; Watcharasit et al. 2002). Therefore, identifying the functional correlation between SIRT1 and GSK3β is important for the development of novel therapies for neuroblastoma.

Here, we first proposed the reciprocal roles of SIRT1/AROS and GSK3β in cytotoxicity in response to DOX treatment. Our data demonstrate that DOX-induced apoptosis was enhanced by deficiency of SIRT1 and AROS and increased by GSK3β expression in human neuroblastoma SH-SY5Y cells. We also identified GSK3β as a binding partner of SIRT1 and AROS. The interaction between GSK3β and SIRT1/AROS was determined in the nucleus by IP and confocal microscopy. GSK3β is mainly known as a cytoplasmic protein, but is also found in the nucleus and mitochondria (Bijur and Jope 2003). Recent studies have shown that nuclear localization of GSK3β is regulated by mTOR complex 1 (mTORC1) (Bautista et al. 2018), and nuclear GSK3β facilitates acute myeloid leukemia (AML) cell growth and drug resistance (Ignatz-Hoover et al. 2018). However, the regulation of nuclear GSK3β is not well defined. Our results suggest that SIRT1 might be a potential regulator of nuclear GSK3β. Furthermore, SIRT1 and AROS inhibited GSK3β by increasing inhibitory phosphorylation on Ser9. Finally, we observed that SIRT1 and AROS deacetylated GSK3β. Since GSK3β binds to SIRT1 and AROS in the nucleus, it is expected to be deacetylated in nucleus. SIRT1-mediated deacetylation of GSK3β may promote inhibitory phosphorylation of GSK3β on Ser9. Recent evidence shows that high glucose induces HDAC1-mediated deacetylation and enhances phosphorylation of S6 kinase in mesangial cells (Das et al. 2019). In addition, a structural basis study suggests that lysine acetylation disrupts phosphorylation in a kinase motif by inhibiting salt bridge (Parker et al. 2014). Therefore, SIRT1-mediated deacetylation of GSK3β is likely to facilitate its inhibitory phosphorylation and may inhibit GSK3β activity. Overall, our results suggest that SIRT1 and AROS inhibit GSK3β activity and provide a basis for the mechanism of DOX efficacy in brain tumor chemotherapy.
